# Health systems strengthening to optimise scale-up in global mental health in low- and middle-income countries: lessons from the frontlines – RETRACTION

**DOI:** 10.1017/S2045796020000311

**Published:** 2020-04-29

**Authors:** I. Petersen, A. van Rensburg, S. Gigaba, Z. P. B. Luvuno, L. Fairall

**Affiliations:** Centre for Rural Health, University of KwaZulu-Natal, Durban, South Africa; Knowledge Translation Unit, University of Cape Town, Cape Town, South Africa; King's Global Health Institute, King's College London, London, UK

The authors would like to retract this paper on the basis that the correct South African Department of Health protocols for communications relating to this body of work were not followed. Their full statement is as follows:

We (the co-authors) have discussed the article with the Department of Health and a collective decision has been reached that we would like the entire article retracted on the basis that some of the information on MhINT that was divulged had overstated the scope covered through MhINT for Department of Health and should thus should not have been included – making aspects of the MhINT content of the editorial currently inaccurate and potentially misleading.
